# Hydrothermal Treatment to Enhance Supercritical CO_2_ Polycaprolactone Foaming Processes for Tissue Engineering Scaffolds

**DOI:** 10.3390/polym17223076

**Published:** 2025-11-20

**Authors:** Belén García-Jarana, Diego Valor, Ignacio García-Casas, Jezabel Sánchez-Oneto, Casimiro Mantell, Juan R. Portela, Clara Pereyra

**Affiliations:** Department of Chemical Engineering and Food Technology, Faculty of Sciences, Wine and Agrifood Research Institute (IVAGRO), University of Cadiz, 11510 Cadiz, Spain; belen.garcia@uca.es (B.G.-J.); ignacio.casas@uca.es (I.G.-C.); jezabel.sanchez@uca.es (J.S.-O.); clara.pereyra@uca.es (C.P.)

**Keywords:** scaffolds, polycaprolactone, polymers, CO_2_, foaming

## Abstract

Hydrothermal treatment was investigated as a strategy to enhance the supercritical CO_2_ foaming process for the fabrication of polycaprolactone (PCL) scaffolds intended for tissue engineering applications. PCL samples were subjected to supercritical foaming at 300 bar and 40 °C for 60 min, combined with hydrothermal treatments performed either before or after foaming at temperatures of 70–100 °C and pressures of 10–20 bar. The effects of these treatments on scaffold morphology, porosity, and mechanical behavior were evaluated using scanning electron microscopy, micro-computed tomography, and compression testing. The results showed that hydrothermal treatment prior to foaming significantly improved scaffold porosity from 16.5% (untreated PCL) up to 57.9% while increasing pore interconnectivity (up to 156.8 throats mm^−3^). Conversely, post-foaming hydrothermal treatment led to pore collapse and loss of structural integrity. The pre-treated scaffolds maintained compressive moduli within 2–12 MPa, consistent with values required for bone tissue engineering. In vitro degradation in PBS revealed a moderate increase in weight loss (~10% after 90 days), indicating that the hydrothermal step slightly accelerates polymer hydrolysis without compromising stability. These findings demonstrate that combining hydrothermal pre-treatment with supercritical CO_2_ foaming provides a solvent-free route to tailor scaffold morphology and mechanical performance, offering a sustainable alternative for the design of bioresorbable materials in regenerative medicine.

## 1. Introduction

Biodegradable materials are increasingly replacing biostable materials in biomedical applications, particularly in tissue engineering, due to their excellent biocompatibility and ability to degrade in physiological environments. These polymeric biomaterials can be classified into hydrolytically and enzymatically degradable materials based on their degradation mechanism, with most synthetic polymers undergoing hydrolytic degradation due to their biologically inert nature. Hydrolytic degradation primarily affects functional groups such as esters, amides, anhydrides, and carbonates, with polyester biomaterials being characterized by their tunable mechanical and thermal properties, as well as their reproducibility [1].

Tissue engineering involves designing three-dimensional scaffolds that can temporarily replace the extracellular matrix. These scaffolds support cell adhesion, proliferation, and differentiation until new tissue forms. Polymeric materials such as PCL and PLA have been widely studied for this purpose because of their tunable degradation rates, biocompatibility, and ability to be processed into porous architectures. PCL in particular offers a favorable balance between mechanical strength and long-term degradability, making it suitable for bone, cartilage, and vascular regeneration applications. The microstructure of these scaffolds, particularly their porosity, pore size, and interconnectivity, plays a decisive role in regulating nutrient transport, vascularization, and mechanical integration with host tissue. Therefore, developing environmentally friendly processing routes that can be used to tailor these structural parameters remains a major focus in the fabrication of polymer-based scaffolds for tissue engineering.

Traditionally, polymer processing for scaffold manufacturing has relied on volatile organic solvents. However, these methods have several drawbacks, including the difficulty of completely removing solvent residues, environmental hazards due to solvent emissions, and the high temperatures required in many processes. In addition, the morphological properties of scaffolds produced using solvent-based methods are often inadequate, particularly in terms of achieving a highly interconnected porous structure with a uniform pore-size distribution [2,3].

To overcome these limitations, supercritical CO_2_ foaming has become progressively more popular due to its success in producing functional scaffolds. This process relies on the unique physical properties of supercritical carbon dioxide that create a porous structure in the polymer for tissue engineering with tunable properties [4]. In addition to the low toxicity of scCO_2_, it is cost-effective, reusable, and avoids the use of organic solvents that can interfere with the polymers [5]. In the first step, the polymer is saturated with CO_2_ at a constant pressure and temperature. The dissolved CO_2_ facilitates the mobility of the polymer chains, lowers the glass transition temperature, and promotes plasticization [6]. The system is then brought to a supersaturated state, usually with a rapid reduction in pressure, although this can also be due to a sudden increase in temperature. A phase separation is produced, which induces cell nucleation by reducing the solubility of CO_2_. Finally, cell growth takes place within the polymer matrix, gradually forming the foam structure [7].

This foaming process requires the polymer to have a high affinity for CO_2_. In this regard, polymers such as PLA, PGA, PLGA, and PCL are among the most widely used materials for tissue engineering scaffolds due to their excellent biocompatibility, biodegradability, suitable mechanical properties, and non-toxic degradation products, as well as their relatively high affinity for CO_2_, making them particularly suitable for processing using supercritical foaming techniques [8,9]. PCL is a semi-crystalline aliphatic polyester with a glass transition temperature (Tg) close to −60 °C and a low melting point of 55–60 °C [10]. Moreover, PCL has a slower degradation rate compared to other polymers, which makes it more suitable for controlled release systems and long-term degradable implants [11]. Therefore, PCL has been widely used for medical purposes; for example, as nanofibers loaded with antibiotic drugs for controlled release [11] and with therapeutic molecules such as proteins [12,13]. On the other hand, this polymer can be used as an additive in resins to improve their resistance or to coat stainless steel against corrosion [14]. Furthermore, PCL has been successfully employed as a scaffold for tissue repair in cardiovascular, nerve, skin, cartilage, and bone engineering [15].

An effective scaffold should provide the structural support and the porous morphology required for cell adhesion and consequent tissue regeneration. In this sense, the supercritical CO_2_ foaming process allows for tuning of the porosity and pore size by adjusting the main foaming parameters (temperature, pressure, CO_2_ contact time, and depressurization gradients) [16,17]. However, as the pore formation mechanisms are complex, it is difficult to obtain precise and predictable control of the pore sizes and distributions in the produced scaffolds, which causes certain limitations in the supercritical process [18]. Therefore, the use of various types of pore-forming substances (e.g., bicarbonates, polyethylene oxide, sodium chloride, sucrose) has been studied in scCO_2_ foaming processes to obtain a well-defined porosity and a good pore size distribution [19,20,21,22]. The type of porogen used, as well as its content and size, has a significant effect on the properties of the produced solid foams. Kosowska et al. analyzed hydroxyapatite, carboxymethylcellulose, nanocellulose, and graphene oxide as porogens, recommending the process of PCL foaming with 5% hydroxyapatite and 0.2% or graphene oxide using scCO_2_ [23]. Ammonium bicarbonate has also been used as a porogen in PCL scaffolds prepared using supercritical foaming [18]. This substance, when incorporated in scaffold formulation, produces a dual porosity that is advantageous for regenerative medicine purposes. Nevertheless, an extra stage is needed to remove the porogen. This step is normally carried out using solvent leaching, usually water. Hence, in the case of drug-loaded scaffolds, porogen removal can reduce the drug load due to leaching of the bioactive substance incorporated into the scaffold formulation [18,24].

Therefore, new strategies are needed to promote the development of interconnected porous networks and the optimal values of the mean pore size without the incorporation of solid porogens. In this context, a hydrothermal treatment could favor these porous structures. This process involves using water at high pressure and temperature. The hydrothermal method is an efficient technique for the synthesis of crystals of hydroxyapatite with a uniform morphology and high crystallinity [25]. The defect-free crystals obtained through hydrothermal processes present a crystallinity with a narrow particle size distribution [26]. Hydrothermal treatment has also been investigated as an efficient technique for modifying polymer structures without the need for additives or cross-linking agents, making it an attractive strategy for tissue engineering applications [27]. In this context, Wasupalli et al. demonstrated that increasing the temperature during hydrothermal treatment enhanced the porosity and interconnectivity of chitosan–polygalacturonic acid polyelectrolyte complex fibrous scaffolds, which are key characteristics for promoting cell adhesion and tissue regeneration [28]. These findings suggest that hydrothermal treatment could be a valuable tool for optimizing the morphology of PCL scaffolds without the need for additional solid porogens, aligning with the objectives of the present study. Moreover, a higher swelling capacity and stiffness were obtained with this hydrothermal treatment in the scaffolds produced for bone tissue engineering. Meanwhile, cellulose nanocrystals hydrogels have also been prepared using hydrothermal treatment [29]. In this study, it was concluded that these hydrogels can be dried to produce an effective scaffold system, supporting their use in different applications.

Although the use of high temperatures is often considered a drawback in conventional foaming or solvent-based processes due to the potential degradation of polymers and the need for additional purification steps, hydrothermal treatment represents a more environmentally friendly alternative. In this case, water at an elevated temperature and pressure acts as a clean and non-toxic medium, avoiding the use of organic solvents or porogens. Under these conditions, temperature plays a beneficial role by inducing chain mobility and partial recrystallization, which can promote the development of interconnected porous structures during subsequent foaming. There is evidence in the literature that higher hydrothermal temperatures improve porosity and interconnectivity in chitosan-based scaffolds and that hydrothermal gelation of cellulose nanocrystals produces stable porous networks [28,29]. Based on this evidence, the hydrothermal conditions used in this study (temperature, pressure, and time) were selected to exceed the glass transition temperature of PCL and approach its melting range [10] without complete melting, thus promoting structural rearrangements favorable for scaffold formation.

Currently, there are no studies on the effects of hydrothermal treatment on the fibrous structure of PCL. In order to take advantage of the scCO_2_ foaming process, which promotes a structural support with a porous morphology necessary for cell adhesion and consequent tissue regeneration without the incorporation of solid porogens, the present work proposes the combination of both processes. Therefore, this study analyzed the effects of performing a hydrothermal treatment before or after the supercritical CO_2_ foaming process on pore size and distribution, and the interconnected porous networks in the PCL produced. In addition, the effect of the thermal treatment on the mechanical properties of the PCL scaffolds was investigated.

## 2. Materials and Methods

### 2.1. Materials

Polycaprolactone was provided by Sigma–Aldrich (Steinheim, Germany) as pellets (average Mw 45.000 g∙mol^−1^). CO_2_ with a minimum purity of 99.8% for the foaming experiments and N_2_ for the hydrothermal treatment were supplied by Linde (Barcelona, Spain). NaCl, KCl, Na_2_HPO_4_, and KH_2_PO_4_ (buffer solution) were provided by Sigma–Aldrich (Steinheim, Germany).

### 2.2. Supercritical CO_2_ Foaming Process

**Figure 1:**
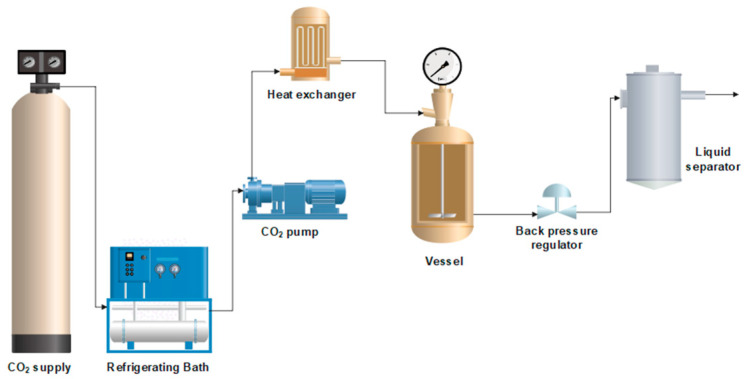
Schematic diagram of the foaming pilot plant.

Polymer foaming was carried out in a pilot plant developed by Thar Technologies (Pittsburgh, PA, USA). A schematic diagram of the pilot plant is shown in Figure 1. The unit consisted of a 257 mL stainless steel vessel in which the foaming was carried out. The apparatus is also equipped with a high-pressure pump, which fills the vessel with CO_2_ in the liquid state up to the required pressure. In order to reach this liquid state, the CO_2_ has been previously cooled through a thermal batch. A heat exchanger is also used to maintain the temperature. Finally, a manual valve is used to control the rate at which the CO_2_ is vented.

Supercritical CO_2_ foaming exposes the polymer to carbon dioxide, which plasticizes the polymer by lowering the glass transition temperature (Tg). In the depressurization step, thermodynamic instability leads to oversaturation of the carbon dioxide dissolved in the polymer matrix, resulting in cell nucleation and the subsequent formation of a larger structure and increased porosity.

First, 0.5 g of PCL was placed into a steel mesh, which was then introduced into the foaming vessel. Later, CO_2_ was pumped into the vessel at the desired conditions. These experiments were carried out at a pressure of 300 bar, temperature of 40 °C, and constant contact time of 60 min (based on previous work [5]). Once the test finished, the outlet valve was opened at a rapid depressurization range of 25–30 bar/min to vent the CO_2_.

### 2.3. Hydrothermal Treatments

Two different types of laboratory-scale equipment were used for the application of the hydrothermal treatment. The first was used for treatment at atmospheric pressure. It consists of a 1 L beaker, a hot plate with stirring, and temperature control from the commercial company SELECTA (Cham, Switzerland). In this treatment, the contact time and the operating temperature were analyzed. The sample was introduced into a stainless-steel basket that was submerged in a glass that was filled with 800 mL of distilled water, which was used to keep the desired temperature constant for a specified period of time.

**Figure 2:**
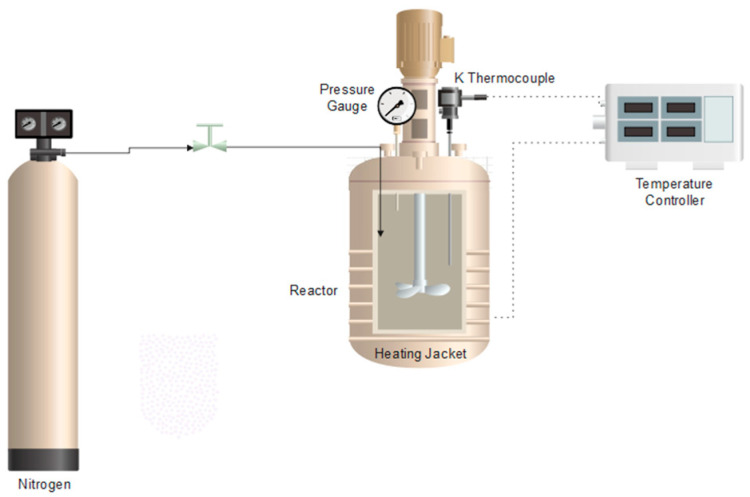
Schematic diagram of the batch reactor for the hydrothermal treatment.

The treatments at high pressure and in the vapor phase were carried out in a batch reactor. A schematic drawing of this device is represented in Figure 2. The equipment consists of a 284 mL volume 316 stainless steel cylindrical reactor (Autoclave Engineers, Erie, PA, USA), which is 21 cm long and 6 cm in diameter. This reactor also has a variable speed stirrer and an electric furnace that controls the temperature using an electronic controller (PID). The procedure, depending on whether the test is carried out in the vapor or submerged phase, consisted of adding 60 or 180 mL of distilled water, respectively, along with the PCL sample, into a stainless-steel basket that is attached to the top of the reactor. These experiments were carried out without stirring. Then, the reactor was closed, and N_2_ was introduced until the desired initial pressure was achieved. Subsequently, the system was heated to the operating temperature, and once it was reached, the temperature and pressure were maintained for the established amount of time. The variables that were controlled were pressure, temperature, and polymer/water contact time.

As mentioned above, the hydrothermal process for the enhancement of CO_2_ foaming was performed before and after the supercritical foaming process. The conditions for applying the hydrothermal process after the usual supercritical foaming process are shown in Table 1. The process temperature, pressure, and time were varied to determine how they affect the process.

The process was then studied in reverse: the hydrothermal treatment was performed before the polymer foaming process in supercritical CO_2_. In this case, taking into account that the process is carried out before the treatment with CO_2_, which decreases the Tg of the polymers, the conditions used were higher, especially in terms of the temperature used (70–100 °C). The pressure, mode of contact, and time variables were selected based on the results obtained in the experiment. A summary of the conditions for applying the hydrothermal process before foaming can be found in Table 2.

### 2.4. Sample Characterization

#### 2.4.1. Scanning Electron Microscopy

In order to examine the morphology of the scaffolds, scanning electron microscopy (SEM) was employed. The microscope used was the Nova NanoSEM 450TM model from the Central Services for Scientific Research and Technology (SC-ICYT) of the University of Cádiz (Cádiz, Spain). PCL samples treated with supercritical CO_2_ and different hydrothermal treatments were coated with a 10 nm film of gold to improve their conductivity. A cross-section of each sample was selected.

#### 2.4.2. Tomography Analysis: Porosity, Connectivity, and Expansion Degree

Micro X-ray computed tomography (µ-CT) scans were conducted using a Zeiss Xradia 610 Versa (Jena, Germany) to investigate the effect of hydrothermal pre-treatment on the formation of PCL scaffolds. The scans were performed at a voxel resolution of 14.89 µm. The 3D images obtained were reconstructed and analyzed using DragonFly version 2022.2 from Object Research Systems (ORS). First, a representative region of interest (ROI) (selected area where foam and porosity were achieved) was isolated to evaluate the effect of the different working parameters on scaffold morphologies. The ROI was then segmented to distinguish the void fraction (pores and pore connections or throats) from the solid material. This segmentation is critical for 3D modeling as it influences the subsequent analyses and structure plots. A workflow known as Pore Network Modeling (OpenPNM) [30] was then employed to determine the number of vertices (pores) and edges (throats) in the selected ROI. Throat length and number of throats are key parameters that describe the connectivity and geometry of a material’s pore network. To calculate them, a pore network analysis based on the segmentation and skeletonization of micro-computed tomography (µ-CT) images was used, employing Dragonfly software (version 2022.2). This method identifies the centers of the pores and the “necks” or throats that connect them, providing a representation of the network as a graph. The throat length is defined as the distance between the centers of two adjacent pores along the channel connecting them, while the number of throats is the total count of these connections in the analyzed volume.

#### 2.4.3. Mechanical Properties

Young’s modulus was calculated to investigate the mechanical durability of the scaffolds [31]. It was determined using compression testing and calculated from the slope of the initial linear portion of the strain curve, or the pressure value divided by the initial surface area of the scaffold. Compression tests were performed using an MTS Criterion C45 testing machine (Eden Prairie, MN, USA). According to the manufacturer’s specifications, the system provides force and strain accuracies of ±0.5% of the applied load and measured value. Each experimental condition was tested in duplicate. The scaffolds were compressed to a total strain of 70%, at a compression rate of 0.02 mm/s and a maximum load of 10 kN. The size of the samples was adjusted to 15 mm^3^ prior to analysis. The mechanical properties of the two processes, i.e., with and without hydrothermal treatment before and after foaming, were studied.

#### 2.4.4. Degradation Tests

The degree of weight loss of the processed polymers was studied in a buffer solution (PBS solution (pH 7.4) containing 8.00 g of NaCl, 0.20 g of KCL, 1.44 g of Na_2_HPO_4_, and 0.20 g of KH_2_PO_4_ in 1L MiliQ water). In the long-term study, at each time point (15, 30, 45, 60, 75, and 90 days), the samples were first removed from the buffer solution, and any excess water was blotted away with filter paper after rinsing with distilled water. Subsequently, the samples were then incubated at 37 °C for 24 h, after which the weight loss was determined according to Equation (1):

(1) $$Weight\;loss\;\left(\%\right)=\;\frac{Wi-Wd}{Wi}\;\times 100$$

where *Wi* is the initial weight and *Wd* is the dry weight at each timepoint.

## 3. Results and Discussion

This study is based on a combined treatment system of scCO_2_ foaming and hydrothermal treatment of PCL polymer. The effect of performing a hydrothermal treatment before or after the supercritical CO_2_ foaming process was analyzed in terms of the pore morphology and mechanical properties of the produced scaffolds. Based on the results, the most complex characterization techniques were carried out on experimental samples 1B, 2B, 3B, and 4B (hydrothermal pretreatment), as these samples showed the most promising potential for improving the properties of the polymers.

**Figure 3:**
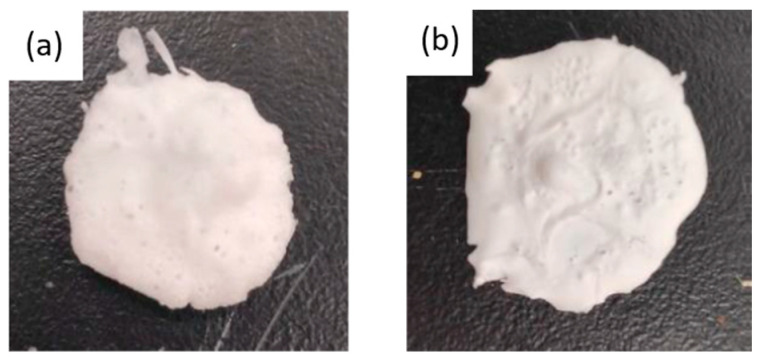
Images of the polymers obtained at 40 °C, a foaming time of 60 min, and (**a**) 300 bar or (**b**) 100 bar.

**Figure 4:**
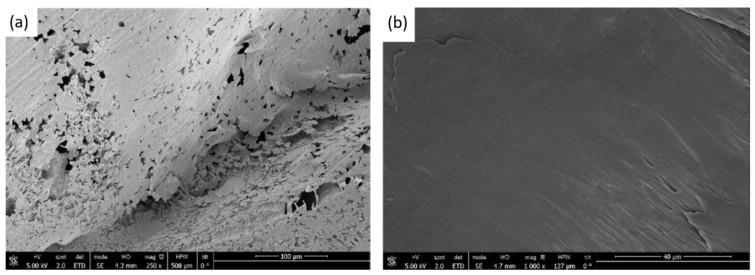
SEM images of PLC scaffolds produced at 300 bar (**a**) and 100 bar (**b**). Scale bars: 100 µm (**a**) and 40 µm (**b**).

The preliminary experiments on scCO_2_ foaming with PLC were carried out at 40 °C with a foaming time of 60 min, as these were determined as the better conditions in a previous study [5]. Similarly, Satpayeva et al. [7] achieved a higher porosity in the scaffolds produced at a lower temperature (40 °C) and with a one-step decompression. As previous studies [6,32] showed a significant effect of pressure on the level of porosity and morphology, two different pressures were analyzed (100 and 300 bar). In Figure 3, images of the scaffolds formed with these pressures are shown. In both cases, the final size of the polymer obtained was very similar. However, according to the SEM images shown in Figure 4, it can be seen that the porosity obtained at a pressure of 300 bar is notably higher than that obtained under the 100 bar condition (porosities of approximately 30% were obtained at the higher pressure). This indicates that higher pressures promote more effective nucleation and pore growth under our processing conditions. Thus, the subsequent foaming experiments were carried out at 300 bar, 40 °C, and for 60 min. These findings are consistent with the trend reported by Chen et al. [32], who also observed that increasing pressure favors porosity up to a certain threshold.

### 3.1. Effect of the Hydrothermal Treatment After Foaming Process

**Figure 5:**
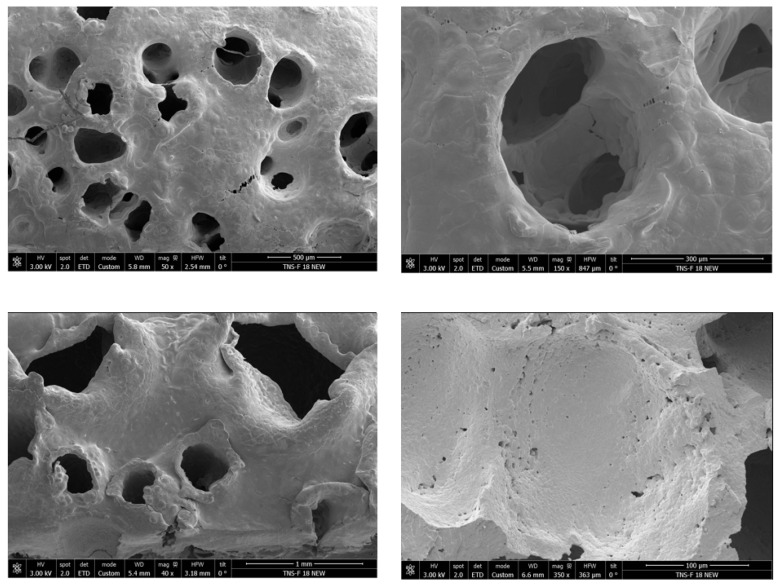
Scanning electron microscopy (SEM) images of the final products from the hydrothermal post-treatment. The image on the right shows sample 1A and the left shows sample 2A.

Once the polymers were treated with CO_2_, the hydrothermal treatment was carried out under different conditions, as shown in Table 2. The application of hydrothermal treatment following the supercritical CO_2_ foaming process adversely affected the morphology and mechanical integrity of the polycaprolactone (PCL) scaffolds. Scanning electron microscopy images (Figure 5) revealed a significant reduction in porosity and pore interconnectivity compared to untreated samples, with noticeable pore collapse and a denser, less porous structure. As a direct consequence, the scaffolds became structurally fragile and could not withstand minimal compressive loads, preventing mechanical testing. This observation highlights the critical importance of maintaining the pore architecture to preserve mechanical stability. Similar behavior has been reported in the literature for highly porous PCL scaffolds, where compression moduli as low as 0.24 MPa have been recorded [33], supporting the relationship between excessive porosity and loss of strength. These findings suggest that post-foaming hydrothermal treatment may detrimentally impact the scaffold’s architecture and mechanical properties, limiting its potential application in tissue engineering, where both porosity and mechanical resilience are crucial.

Given the structural instability and poor morphological properties observed in the scaffolds subjected to post-foaming hydrothermal treatment, further analysis of these samples was deemed unfeasible. Therefore, the discussion will now center on the impact of pre-foaming hydrothermal treatment on scaffold morphology, porosity, interconnectivity, and mechanical performance, as these conditions have shown greater potential for generating structurally robust and well-defined porous architectures suitable for tissue engineering applications.

### 3.2. PCL Porosity, Pore, and Connectivity Analyses

As previously mentioned, the polymeric matrices formed by PCL were produced using a two-step process. The first step involved a hydrothermal treatment conducted at varying temperatures and pressures (Table 3), followed by a foaming process using supercritical CO_2_ under consistent operating conditions of 300 bar, 40 °C, and 60 min. The objective of this part of the study was to investigate the impact of the initial hydrothermal treatment on the morphology of the resulting scaffold. The key characteristics of macropores ranging from 100 to 1000 µm and good interconnectivity are crucial for achieving an optimal scaffold structure [34]. A sample of PCL treated only with CO_2_ was also analyzed as a control.

**Figure 6:**
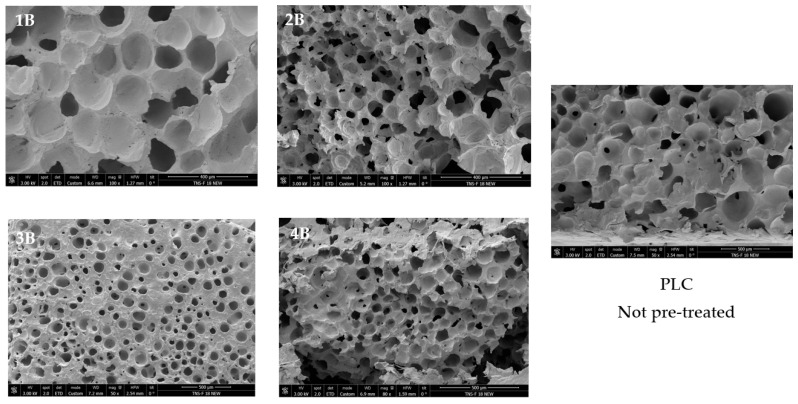
SEM images of the samples subjected to hydrothermal pre-treatment. Scale bars: 100 µm (1B), 400 µm (2B), 500 µm (3B), 500 µm (4B), and 500 µm (PCL not pre-treated).

The SEM analysis (Figure 6) revealed notable differences in the porous morphology between the pretreated samples (1B, 2B, 3B, and 4B) and the untreated PCL scaffold. The hydrothermally treated samples exhibit a higher number of pores that were distributed more homogeneously throughout the structure compared to the untreated PCL, which presented a less defined and more irregular pore distribution. Additionally, the pretreated scaffolds displayed a more ordered architecture, suggesting an enhanced control over the foaming process due to the hydrothermal step. This structural organization hints at a potentially higher degree of pore interconnectivity, which was further examined in the following part through X-ray microtomography analysis.

**Figure 7:**
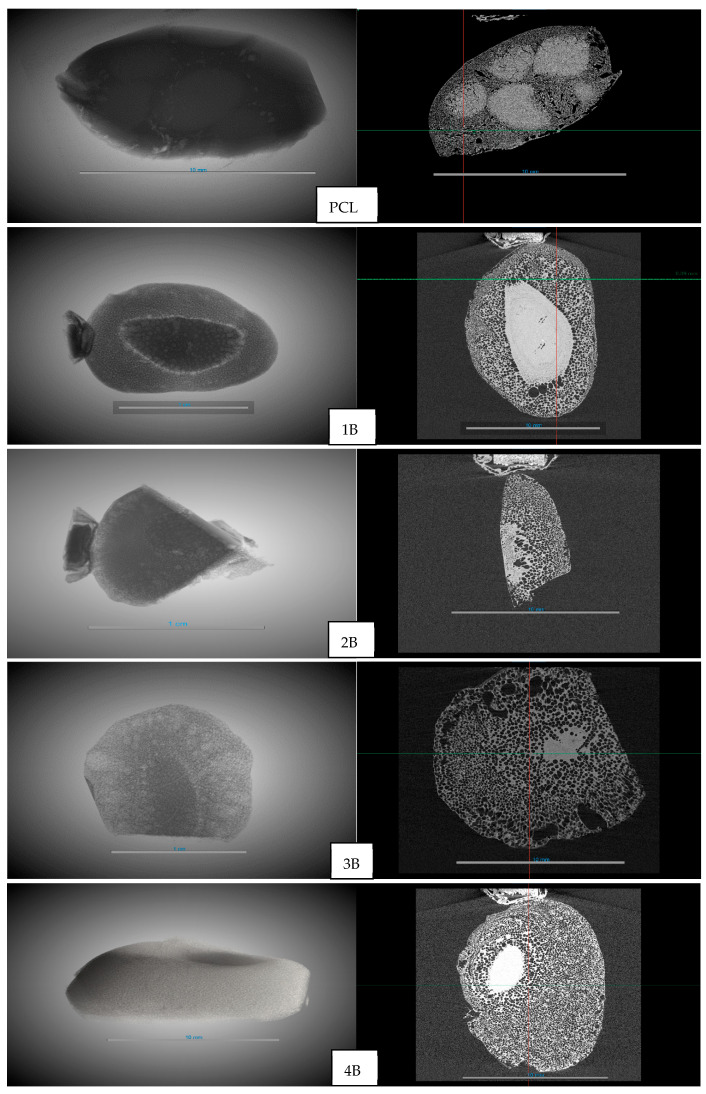
Three-dimensional (**left**) and YZ cross-section (**right**) images of foaming polymers. Scale bar: 10 mm (all samples).

Based on the results shown in Table 3, a significant influence on the porosity of the scaffolds was observed for the initial hydrothermal treatment, with the porosity increasing from 16.54% in the untreated PCL to a range of 41.67–57.90%. Figure 7 shows the 3D reconstruction of the formed frameworks and the YZ cross-section. In the PCL without hydrothermal treatment, it can be seen that the foaming process with supercritical CO_2_ was less effective, with visible areas where the PCL had undergone foaming (white areas). In the other cases, with prior hydrothermal treatment, both the 3D images and the YZ cross-sections showed that the areas where the PCL had not undergone foaming were significantly smaller.

The apparent discrepancy between the overall porosity and pore density values can be explained by pore coalescence occurring at higher porosity levels. In these circumstances, neighboring pores tend to merge, creating larger interconnected voids that boost the total void fraction while reducing the number of individual pores per unit volume. This phenomenon is typical of supercritical CO_2_ foaming processes and indicates structural coalescence rather than sampling inhomogeneity.

**Figure 8:**
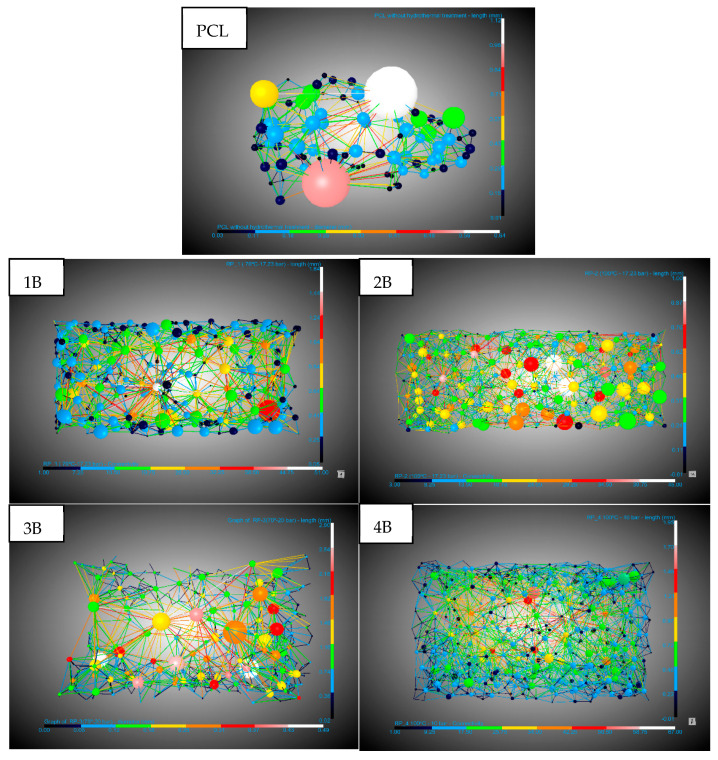
DragonFly images of PCL foaming experiments without and with pre-hydrothermal treatment. Y−bar shows throat length scale. X−bar shows pore diameter scale.

The areas of interest in the scaffolds where foaming and pore formation had occurred were analyzed. The main aspects of a scaffold, such as its pores and interconnectivity, were studied (Figure 8 and Table 3). There was a slight increase in pore diameter in the scaffolds generated with hydrothermal treatment, from an average of 0.12 mm for the untreated PCL to 0.17 mm for the scaffold whose pre-treatment was at the highest temperature (100 °C) and an average pressure of 17.23 bar. In terms of the pore density, a decrease was observed compared to untreated PCL. This reduction is likely associated with the combined effect of a lower nucleation site density and increased pore growth during the foaming process, resulting in fewer but larger pores within the scaffold structure. On the other hand, the connectivity decreased in samples 1B, 2B, and 4B, which also had the longest channel lengths (0.49–0.64 mm), while 2B and the untreated PCL showed a greater number of connections with average lengths of 0.29 mm and 0.34 mm, respectively. A good scaffold is defined by its porosity, pore density, and connectivity. The 2B experiment, with hydrothermal pre-treatment at the highest temperature (100 °C) and a pressure of 17.23 bar, shows a three-times higher porosity than that of the untreated PCL but also maintained a similar pore density (27.8 vs. 30.6 pores/mm^3^) and high connectivity (156.8 vs. 126.7 throats/mm^3^) compared to the small porous regions in the untreated PCL. This demonstrates the potential of hydrothermal pre-treatment in significantly enhancing the design of PCL scaffolds, offering a balance between increased porosity and preserved connectivity.

It should be noted that the micro-CT images presented correspond to individual slices of the reconstructed volume. Therefore, the compact white regions do not necessarily extend along the entire axis of the sample, but rather represent localized areas without foaming. The appearance of these regions has been previously described in PCL foaming with supercritical CO_2_ [17] and is associated with diffusion limitations and heterogeneous nucleation. However, the hydrothermal pretreatment significantly reduced the presence of these compact domains, resulting in scaffolds with more uniformly distributed pores.

### 3.3. Mechanical Properties

**Figure 9:**
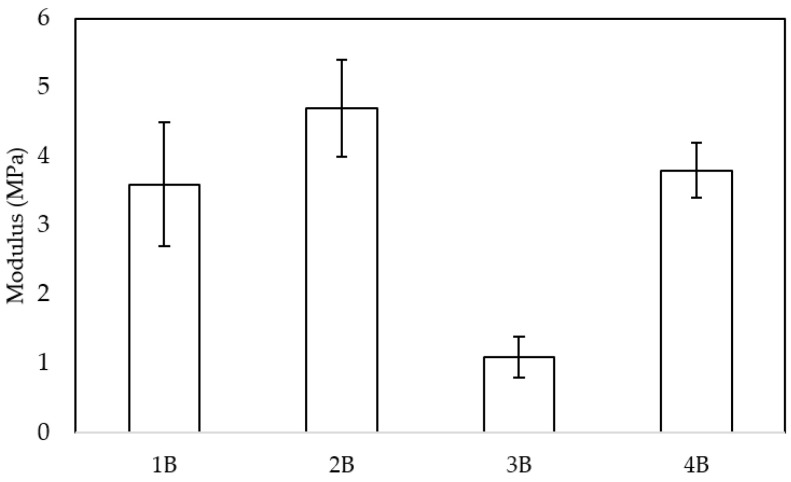
Compressive properties of the PCL pre-treated scaffolds.

Figure 9 presents the compression modulus of the four scaffold samples. Notably, sample 2B exhibited the highest compression modulus, which aligns with its superior interconnectivity (156.8 throats/mm^3^) as determined by the X-ray tomography analysis. This result is partially consistent with previous studies. For example, Murphy et al. [35] demonstrated that increased pore interconnectivity can significantly alter mechanical performance, although the effect may depend on the processing route. In their work, solvent-molded scaffolds showed an increase in modulus with higher interconnectivity, while gas-foamed scaffolds showed the opposite trend. In our case, the higher interconnectivity of sample 2B appears to contribute to better stress distribution and a higher compressive modulus. In addition, Carr et al. [36] indicated an inverse relationship between these two parameters, obtaining a decrease in the elastic modulus as porosity increased. Conversely, sample 3B, which had the highest porosity (57%) compared to the other samples, demonstrated the lowest compression modulus. This discrepancy may be attributed to the lower interconnectivity and pore density per unit volume in 3B, which implies the presence of larger pores. Larger pores can lead to reduced structural integrity, as the load-bearing capacity of the scaffold is weakened due to fewer supporting struts within the microstructure. Thus, while porosity plays a crucial role in scaffold architecture, the balance between pore size, density, and interconnectivity appears to be a key determinant of mechanical performance.

When assessing the mechanical properties of a material, both stiffness and strength are key factors, as their values can differ substantially based on the intended implantation site and its specific mechanical demands. In the field of bone tissue engineering, various studies have reported compression strength values in the range of 2–12 MPa for scaffolds fabricated using different techniques [37,38,39]. These values serve as a reference for evaluating the mechanical feasibility of newly developed materials. Given this context, the mechanical performance of the pretreated samples in this study falls within an acceptable range, supporting their potential suitability for specific applications where similar mechanical properties are required. It is important to note that the mechanical properties of PCL scaffolds can vary significantly depending on the manufacturing technique used, the porosity, and the incorporation of other materials or treatments.

Although porosity is a key parameter that influences the mechanical behavior of scaffolds, the distribution of different pore sizes (polydispersity) and the presence of large defects can have a greater impact on strength than macroscopic porosity alone, particularly at low-to-moderate porosity levels. This was examined in the present study. Local heterogeneities and coalesced large pores act as stress concentrators, reducing the effective load-bearing cross-section. Consequently, samples with similar overall void fractions can exhibit markedly different compressive moduli when they differ in defect size and pore-size distribution [40].

Conversely, pore interconnectivity and an evenly distributed network of throats can improve load transfer through the scaffold by offering several load paths and encouraging a more consistent stress distribution. This observation helps to explain why, despite its elevated porosity, sample 2B exhibited relatively high connectivity and the highest compression modulus. In other words, the mechanical responses of our samples appear to be governed by a balance between (i) the reduction in strength caused by large, inhomogeneous defects, which are dominant in the most porous or coalesced structures; and (ii) the mechanical reinforcement derived from a well-connected pore network, which distributes stresses more evenly [41].

Therefore, porosity alone is not a sufficient predictor of compressive performance. To understand the mechanical differences between samples with comparable global porosity, the combined descriptors of pore size distribution, maximum defect size, and connectivity must be considered. A more detailed statistical analysis of defect size distributions and larger replicate numbers would clarify the relative importance of these factors using the current process [42].

### 3.4. In Vitro Degradation Studies

**Figure 10:**
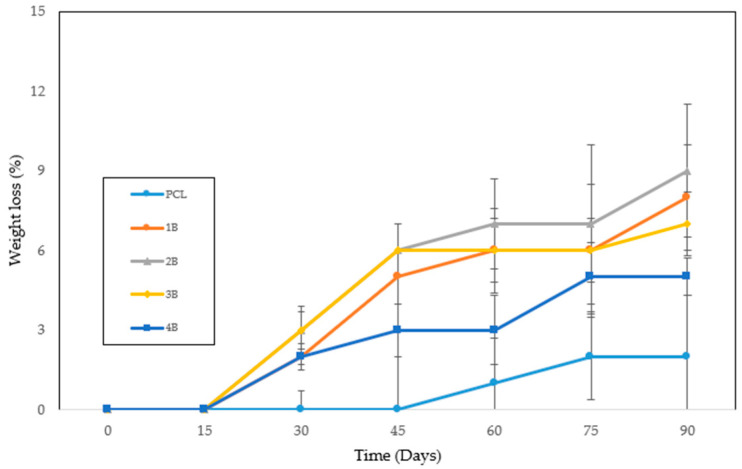
Degradation test of materials treated with hydrothermal and foaming processes and untreated polycaprolactone.

The in vitro degradation of polycaprolactone (PCL) scaffolds was assessed in phosphate-buffered saline (PBS) over a period of 90 days. Five different samples were studied: untreated PCL and four hydrothermally treated PCL samples (1B, 2B, 3B, and 4B) processed at temperatures ranging from 70 °C to 100 °C. The results (Figure 10) indicate a slight increase in weight loss for the hydrothermally treated samples compared to the untreated PCL, suggesting that hydrothermal processing may influence the degradation behavior.

PCL is known for its slow degradation due to its high hydrophobicity and semicrystalline nature, with degradation mainly driven by hydrolytic cleavage of ester bonds. Studies consistently report that degradation rates are closely linked to polymer crystallinity, as crystalline domains hinder water penetration and enzymatic accessibility, while amorphous regions are more prone to hydrolysis [1]. In our study, the moderate increase in weight loss observed for hydrothermally treated samples suggests that the treatment may induce subtle modifications to the crystalline organization of PCL [43], thereby facilitating water uptake and accelerating hydrolytic attack. Although techniques such as FTIR or XRD could provide complementary confirmation at the molecular and structural levels, early-stage degradation often produces changes too subtle to be reliably detected within the 90-day window analyzed. Instead, the combined evidence from the morphological analysis, porosity characterization, and in vitro degradation behavior provides a robust indication that hydrothermal treatment alters scaffold microstructure in a way that impacts crystallinity and, consequently, degradation dynamics. In any case, there do not appear to be any significant differences between the treated samples, as can be seen in the overlap of the error bars at different times.

It should be noted that porosity and interconnectivity are not only crucial for internal tissue growth but also directly affect mass transport properties, such as nutrient diffusion and drug-loading capacity. Previous studies have shown that scaffold porosity regulates drug release kinetics and facilitates cellular nutrient transport within the 3D matrix [17,33]. In this work, we focused on the structural and physicochemical characterization of PCL scaffolds, which represent a necessary first step before evaluating their drug-loading and biological performance. Therefore, future research will extend this work towards in vitro drug release and cell compatibility studies in order to validate their potential in tissue engineering applications.

Alternative strategies, such as blending polymers with PLA or using ultrasonic irradiation, have been reported to improve the pore morphology and mechanical properties of PCL scaffolds. However, these approaches often require the incorporation of secondary components, whereas the hydrothermal process represents a solvent- and additive-free alternative that preserves the chemical composition of PCL. Importantly, our degradation results (Figure 10) showed only a slight increase in weight loss of hydrothermally treated samples compared to untreated PCL, indicating that the treatment did not compromise the stability of the polymer. Therefore, hydrothermal pretreatment can be considered an environmentally friendly and scalable method for improving porosity and interconnectivity without the drawbacks associated with the removal of porogens or polymer blending.

## 4. Conclusions

In this study, we investigated the influence of hydrothermal treatment on the morphology and mechanical behavior of polycaprolactone scaffolds produced using supercritical CO_2_ foaming. The main outcomes highlight how pre- and post-foaming hydrothermal steps affect porosity, pore connectivity, and mechanical performance. Specifically, hydrothermal pre-treatment was shown to significantly enhance porosity and interconnectivity while maintaining mechanical integrity. This work demonstrates that hydrothermal treatment before supercritical CO_2_ foaming significantly enhances scaffold properties, while post-foaming treatment compromises structural integrity. Pre-foaming hydrothermal treatment at 373 K and 17.23 bar resulted in scaffolds with a porosity of 51.88%, a pore diameter of 0.17 mm, and a throat density of 156.8 throats/mm^3^, making them more suitable for tissue engineering applications. In contrast, post-foaming hydrothermal treatment led to pore collapse, mechanical weakness, and lower interconnectivity, rendering the scaffolds unsuitable for structural applications. Micro-computed tomography (µ-CT) confirmed an improved pore distribution, enhanced interconnectivity, and reduced non-foamed regions in the scaffolds subjected to pre-foaming treatment. Mechanical testing demonstrated that the pre-treated scaffolds maintained a compressive modulus within the range required for biomedical applications, ensuring their potential use in load-bearing environments. Furthermore, the in vitro degradation study revealed that the hydrothermally treated PCL scaffolds exhibited a slight increase in weight loss compared to the untreated PCL, suggesting that the hydrothermal treatment influences the degradation behavior and crystallinity of the polymer. Additionally, this study suggests that fine-tuning the hydrothermal parameters, such as temperature, pressure, and exposure time, could further optimize scaffold performance. These results highlight the importance of hydrothermal pre-treatment as a scalable and effective strategy for producing highly porous, mechanically stable scaffolds without additional porogens. Future research should investigate the in vitro and in vivo biological responses, scaffold degradation kinetics, and potential functionalization strategies to enhance bioactivity and promote tissue integration in clinical applications.

## Figures and Tables

**Table 1:** Operating conditions of hydrothermal treatment when applied after CO_2_ foaming.

Run	Temperature (°C)	Pressure (bar)	Time (min)
1A	40	1	1
2A	40	1	10
3A	60	1	1
4A	60	1	10
5A	60	17	10

**Table 2:** Operating conditions of hydrothermal treatment when applied before CO_2_ foaming.

Run	Temperature (°C)	Pressure (bar)	Time (min)
1B	70	17.23	10
2B	100	17.23	10
3B	70	20.67	10
4B	100	10	10

**Table 3:** Microporosity analysis results for the experiments conducted at 300 bar, 40 °C, and 60 min (foaming process). These samples (1–4B) underwent hydrothermal pretreatment. HT-T and HT-P indicate the conditions in the hydrothermal test prior to foaming.

Run	HT-T (°C)	HT-P (bar)	Porosity (%)	Pore Diameter (mm)	Pore Density (n° of Pores/mm^3^)	Throat Length (mm)	Number of Throats per mm^3^
1B	70	17.23	41.67	0.13 ± 0.04	18.3	0.49 ± 0.25	86.2
2B	100	17.23	51.88	0.17 ± 0.09	27.8	0.29 ± 0.15	156.8
3B	70	20.67	57.90	0.14 ± 0.08	9.4	0.64 ± 0.45	30.9
4B	100	10	52.72	0.16 ± 0.06	16.2	0.48 ± 0.28	87.4
PCL (not pre-treated)	-	-	16.54	0.12 ± 0.09	30.6	0.34 ± 0.17	126.7

## Data Availability

Data are contained within the article.

## References

[1] Nair L.S., Laurencin C.T. (2007). Biodegradable Polymers as Biomaterials. Prog. Polym. Sci..

[2] Wang M., Xu P., Lei B. (2023). Engineering Multifunctional Bioactive Citrate-Based Biomaterials for Tissue Engineering. Bioact. Mater..

[3] Alegret N., Dominguez-Alfaro A., Mecerreyes D. (2019). 3D Scaffolds Based on Conductive Polymers for Biomedical Applications. Biomacromolecules.

[4] Ivanovic J., Knauer S., Fanovich A., Milovanovic S., Stamenic M., Jaeger P., Zizovic I., Eggers R. (2016). Supercritical CO_2_ Sorption Kinetics and Thymol Impregnation of PCL and PCL-HA. J. Supercrit. Fluids.

[5] García-Casas I., Montes A., Valor D., Pereyra C., Mart E.J., Ossa D. (2019). Foaming of Polycaprolactone and Its Impregnation with Quercetin Using Supercritical CO_2_. Polymers.

[6] Montes A., Valor D., Delgado L., Pereyra C., de la Ossa E.M. (2022). An Attempt to Optimize Supercritical CO_2_ Polyaniline-Polycaprolactone Foaming Processes to Produce Tissue Engineering Scaffolds. Polymers.

[7] Satpayeva A., Rojas A., Tyrka M., Ksepko E., Galotto M.J., Zizovic I. (2022). Supercritical Foaming and Impregnation of Polycaprolactone and Polycaprolactone-Hydroxyapatite Composites with Carvacrol. Processes.

[8] Zhou Y., Tian Y., Peng X. (2023). Applications and Challenges of Supercritical Foaming Technology. Polymers.

[9] Socci M.C., Rodríguez G., Oliva E., Fushimi S., Takabatake K., Nagatsuka H., Felice C.J., Rodríguez A.P. (2023). Polymeric Materials, Advances and Applications in Tissue Engineering: A Review. Bioengineering.

[10] Ajiboye A.L., Trivedi V., Mitchell J.C. (2018). Preparation of Polycaprolactone Nanoparticles via Supercritical Carbon Dioxide Extraction of Emulsions. Drug Deliv. Transl. Res..

[11] Karuppuswamy P., Reddy Venugopal J., Navaneethan B., Luwang Laiva A., Ramakrishna S. (2015). Polycaprolactone Nanofibers for the Controlled Release of Tetracycline Hydrochloride. Mater. Lett..

[12] Jia J., Duan Y.Y., Yu J., Lu J.W. (2008). Preparation and Immobilization of Soluble Eggshell Membrane Protein on the Electrospun Nanofibers to Enhance Cell Adhesion and Growth. J. Biomed. Mater. Res. A.

[13] Mondal D., Griffith M., Venkatraman S.S. (2016). Polycaprolactone-Based Biomaterials for Tissue Engineering and Drug Delivery: Current Scenario and Challenges. Int. J. Polym. Mater. Polym. Biomater..

[14] Kamberli E., Monajjemi M., Kandemirli F., Mollaamin F. (2023). Simulation Study of Poly-Caprolactone, Chitosan, and Vinyl Ester Resin-Coated Stainless Steel to Improve Corrosion Behavior, Bioactivity, and Biodegradability. Biointerface Res. Appl. Chem..

[15] Espinoza S.M., Patil H.I., San Martin Martinez E., Casañas Pimentel R., Ige P.P. (2020). Poly-ε-Caprolactone (PCL), a Promising Polymer for Pharmaceutical and Biomedical Applications: Focus on Nanomedicine in Cancer. Int. J. Polym. Mater. Polym. Biomater..

[16] Baldino L., Cardea S. (2021). Biopolymeric Porous Structures Obtained by Supercritical Fluids Assisted Processes. Chem. Eng. Trans..

[17] Santos-Rosales V., Gallo M., Jaeger P., Alvarez-Lorenzo C., Gómez-Amoza J.L., García-González C.A. (2020). New Insights in the Morphological Characterization and Modelling of Poly(ε-Caprolactone) Bone Scaffolds Obtained by Supercritical CO_2_ Foaming. J. Supercrit. Fluids.

[18] Santos-Rosales V., Ardao I., Goimil L., Gomez-Amoza J.L., García-González C.A. (2021). Solvent-Free Processing of Drug-Loaded Poly(ε-Caprolactone) Scaffolds with Tunable Macroporosity by Combination of Supercritical Foaming and Thermal Porogen Leaching. Polymers.

[19] Wang X., Salick M.R., Gao Y., Jiang J., Li X., Liu F., Cordie T., Li Q., Turng L.S. (2018). Interconnected Porous Poly(ɛ-Caprolactone) Tissue Engineering Scaffolds Fabricated by Microcellular Injection Molding. J. Cell. Plast..

[20] Zhang K., Wang Y., Jiang J., Wang X., Hou J., Sun S., Li Q. (2019). Fabrication of Highly Interconnected Porous Poly(ɛ-Caprolactone) Scaffolds with Supercritical CO2 Foaming and Polymer Leaching. J. Mater. Sci..

[21] Jing X., Mi H.Y., Cordie T., Salick M., Peng X.F., Turng L.S. (2014). Fabrication of Porous Poly(ε-Caprolactone) Scaffolds Containing Chitosan Nanofibers by Combining Extrusion Foaming, Leaching, and Freeze-Drying Methods. Ind. Eng. Chem. Res..

[22] Rodrigues L.R., De Laranjeira M.S., Fernandes M.H., Monteiro F.J., De Carvalho Zavaglia C.A. (2014). HA/TCP Scaffolds Obtained by Sucrose Crystal Leaching Method: Preliminary in Vitro Evaluation. Mater. Res..

[23] Kosowska K., Krzysztoforski J., Henczka M. (2022). Foaming of PCL-Based Composites Using ScCO_2_: Structure and Physical Properties. Materials.

[24] Deng A., Chen A., Wang S., Li Y., Liu Y., Cheng X., Zhao Z., Lin D. (2013). Porous Nanostructured Poly-l-Lactide Scaffolds Prepared by Phase Inversion Using Supercritical CO_2_ as a Nonsolvent in the Presence of Ammonium Bicarbonate Particles. J. Supercrit. Fluids.

[25] Qi Y., Shen J., Jiang Q., Jin B., Chen J., Zhang X. (2015). The Morphology Control of Hydroxyapatite Microsphere at High PH Values by Hydrothermal Method. Adv. Powder Technol..

[26] Neira I.S., Kolen’kO Y.V., Lebedev O.I., Van Tendeloo G., Gupta H.S., Guitián F., Yoshimura M. (2009). An Effective Morphology Control of Hydroxyapatite Crystals via & DESIGN 2009 ND. Cryst. Growth Des..

[27] Nata I.F., Wang S.S.S., Wu T.M., Lee C.K. (2012). β-Chitin Nanofibrils for Self-Sustaining Hydrogels Preparation via Hydrothermal Treatment. Carbohydr. Polym..

[28] Wasupalli G.K., Verma D. (2022). Development of Chitosan-Polygalacturonic Acid Polyelectrolyte Complex Fibrous Scaffolds Using the Hydrothermal Treatment for Bone Tissue Engineering. J. Biomed. Mater. Res. A.

[29] Lewis L., Derakhshandeh M., Hatzikiriakos S.G., Hamad W.Y., MacLachlan M.J. (2016). Hydrothermal Gelation of Aqueous Cellulose Nanocrystal Suspensions. Biomacromolecules.

[30] Gostick J., Aghighi M., Hinebaugh J., Tranter T., Hoeh M.A., Day H., Spellacy B., Sharqawy M.H., Bazylak A., Burns A. (2016). OpenPNM: A Pore Network Modeling Package. Comput. Sci. Eng..

[31] White L.J., Hutter V., Tai H., Howdle S.M., Shakesheff K.M. (2012). The Effect of Processing Variables on Morphological and Mechanical Properties of Supercritical CO_2_ Foamed Scaffolds for Tissue Engineering. Acta Biomater..

[32] Chen C.X., Liu Q.Q., Xin X., Guan Y.X., Yao S.J. (2016). Pore Formation of Poly(ε-Caprolactone) Scaffolds with Melting Point Reduction in Supercritical CO_2_ Foaming. J. Supercrit. Fluids.

[33] Guarino V., Causa F., Ambrosio L. (2007). Porosity and Mechanical Properties Relationship in PCL Porous Scaffolds. J. Appl. Biomater. Biomech..

[34] Dufner L., Oßwald B., Eberspaecher J., Riedel B., Kling C., Kern F., Seidenstuecker M. (2023). Adjustment of Micro- and Macroporosity of ß-TCP Scaffolds Using Solid-Stabilized Foams as Bone Replacement. Bioengineering.

[35] Murphy W.L., Dennis R.G., Kileny J.L., Mooney D.J. (2002). Salt Fusion: An Approach to Improve Pore Interconnectivity within Tissue Engineering Scaffolds. Tissue Eng..

[36] Carr J., Milhet X., Gadaud P., Boyer S.A.E., Thompson G.E., Lee P. (2015). Quantitative Characterization of Porosity and Determination of Elastic Modulus for Sintered Micro-Silver Joints. J. Mater. Process Technol..

[37] Velasco M.A., Narváez-Tovar C.A., Garzón-Alvarado D.A. (2015). Design, Materials, and Mechanobiology of Biodegradable Scaffolds for Bone Tissue Engineering. Biomed. Res. Int..

[38] Fröhlich M., Grayson W.L., Wan L.Q., Marolt D., Drobnic M., Vunjak-Novakovic G. (2008). Tissue Engineered Bone Grafts: Biological Requirements, Tissue Culture and Clinical Relevance. Curr. Stem Cell Res. Ther..

[39] Polo-Corrales L., Latorre-Esteves M., Ramirez-Vick J.E. (2014). Scaffold Design for Bone Regeneration. J. Nanosci. Nanotechnol..

[40] Mukasheva F., Adilova L., Dyussenbinov A., Yernaimanova B., Abilev M., Akilbekova D. (2024). Optimizing scaffold pore size for tissue engineering: Insights across various tissue types. Front. Bioeng. Biotechnol..

[41] Rege A., Aney S., Milow B. (2021). Influence of Pore-Size Distributions and Pore-Wall Mechanics on the Mechanical Behavior of Cellular Solids like Aerogels. Phys. Rev. E.

[42] Aydin M.S., Sahin M., Dogan Z., Kiziltas G. (2023). Microstructural Characterization of PCL-HA Bone Scaffolds Based on Nonsolvent-Induced Phase Separation. ACS Omega.

[43] Dias J.R., Sousa A., Augusto A., Bártolo P.J., Granja P.L. (2022). Electrospun Polycaprolactone (PCL) Degradation: An In Vitro and In Vivo Study. Polymers.

